# Impact of Instrumented Spinal Fusion on the Development of Vertebral Compression Fracture

**DOI:** 10.1097/MD.0000000000003455

**Published:** 2016-04-29

**Authors:** Yen-Chun Chiu, Tsung-Ting Tsai, Shih-Chieh Yang, Hung-Shu Chen, Yu-Hsien Kao, Yuan-Kun Tu

**Affiliations:** From the Department of Orthopedic Surgery, E-Da Hospital/I-Shou University (Y-CC, S-CY, H-SC, Y-HK, Y-KT), Kaohsiung, Taiwan, R.O.C.; and Department of Orthopedic Surgery, Spine Section, Bone and Joint Research Center, Chang Gung Memorial Hospital/Chang Gung University (T-TT), Taoyuan, Taiwan, R.O.C.

## Abstract

Instrumented spinal fusion has become one of the most common surgeries for patients with various spinal disorders. Only few studies have reported subsequent vertebral compression fractures (VCFs) after instrumented spinal fusion. The purpose of this study was to evaluate the risk of new VCFs in patients undergoing instrumented spinal fusion.

We obtained claims data from the National Health Insurance Research Database of Taiwan and retrospectively reviewed 6949 patients with instrumented spinal fusion as the spinal fusion cohort. Control subjects were individually matched at a ratio of 10:1 with those of the spinal fusion cohort according to age, sex, and the index day. Comorbidities were classified as those existing before the index day, and these included diabetes mellitus, hypertension, osteoporosis, and cerebrovascular accident. The end of the follow-up period for the analyses was marked on the day new VCFs developed, enrolment in the National Health Insurance was terminated, on the day of death, or until the end of 2012. We used the Cox proportion hazards model to analyze the hazard ratio (HR) for developing new VCFs.

Patients with instrumented spinal fusion were significantly more likely to develop new VCFs (1.87% vs .25%, HR: 8.56; *P* < 0.001). Female, elderly, and osteoporotic patients had a high incidence of new VCFs after spinal fusion. The HR for developing new VCFs after instrumented spinal fusion was higher in patients younger than 65 years than in those 65 years or older (HR: 10.61 vs 8.09). Male patients with instrumented spinal fusion also had a higher HR of developing new VCFs than female patients (men, HR: 26.42; women, HR: 7.53).

In our retrospective cohort study, patients who had undergone instrumented spinal fusion surgery exhibited an increased risk of developing new VCFs. Particularly, the HR increased in young (age <65 years) and male patients.

## INTRODUCTION

Initially, spinal fusion surgery was performed by placing bone graft along the spine and fusing it in situ. After the operation, prolonged periods of bed rest and immobilization were usually necessary. However, the rate of pseudarthrosis was high (around 45%),^1,2^ even if patients were carefully monitored. In the late 1950s, the modern instrumented spinal fusion technique was developed when the Harrington hook and rod system was introduced.^3^ In 1973, Luque^4^ first introduced segmental instrumentation and revolutionized the instrumented spinal fusion technique in a new era. With advancements in the spinal technique and instrumentation, instrumented spinal fusion has now become one of the most commonly performed surgeries for various spinal diseases.^5,6^ It is used in cases of spinal trauma, tumours, infection, and scoliosis; also, it has been used more frequently to treat degenerative spondylolisthesis and disc-related problems.^7,8^

In previous reports, patients who had undergone spinal fusion had better pain control and functional scores for their spinal problems than those who had not undergone spinal fusion.^7,9^ The development of posterolateral fusion (PLF) with pedicle screw instrumentation offers a much higher fusion rate than noninstrumented fusion alone, which therefore increases patient satisfaction.^7^ As a result, instrumented spinal fusion with a pedicle screw system has become a popular technique in degenerative spinal surgeries, and it is widely used for treating various spinal disorders.^8,10,11^

However, fusion procedures are not the end of the degeneration process. It is not surprising that as more elderly patients undergo these surgeries, more fusion-related complications are found. Disc degeneration, listhesis, instability, facet arthritis, and stenosis relating to spinal fusion have been widely reported in literature reviews.^12–14^ However, vertebral compression fractures (VCFs), another common problem causing pain in patients, have not been well described in the literature. Bogdanffy et al^15^ reported that a decrease of bone mineral density occurs after spinal fusion surgery. The rigid and longer lever arm after spinal fusion can increase stress on the proximal segments, which may further increase the possibility of developing new VCFs. However, the risk of new VCFs after spinal fusion is still not well defined. In the present retrospective cohort study, which was derived from the National Health Insurance Research Database (NHIRD) of Taiwan, we attempted to determine the risk of subsequent new VCFs after spinal fusion surgery.

## PATIENTS AND METHODS

### Source of Data

The National Health Insurance (NHI) program in Taiwan has operated since 1995 and enrolled nearly all inhabitants of the country. The NHIRD at the National Health Research Institutes (NHRI) is currently in charge of the entire database of NHI claims, and it has published numerous extracted datasets for researchers. The NHRI released a cohort dataset composed of 1,000,000 randomly sampled people alive during 2000. This dataset is called the Longitudinal Health Insurance Database 2000 (LHID 2000). The database collected all the records of these individuals from 1997 to the present. Until the end of 2012, all sampled individuals were followed for outcome identification by using the *International Classification of Disease, Ninth Revision, Clinical Modification* (*ICD-9-CM*). This study was exempt from review by our institutional review board (EMRP-101-027).

### Subjects

**FIGURE 1:**
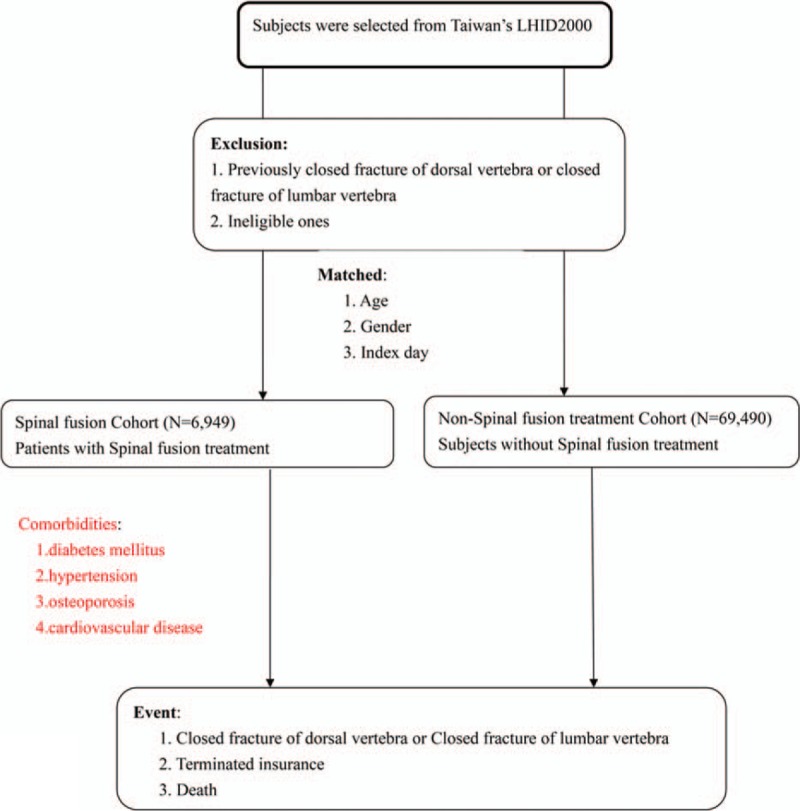
Study flow.

This cohort study used the LHID 2000 to evaluate the risk of closed fracture of the thoracic vertebra (*ICD-9-CM* code 805.2) or closed fracture of the lumbar vertebra (*ICD-9-CM* code 805.4) following instrumented spinal fusion. For the cohort study, 6949 inpatients who had undergone instrumented spinal fusion as first-time treatment from January 1, 1997 to December 31, 2010 were selected and defined as the instrumented spinal fusion cohort. The date of the spinal fusion surgery was assigned as the index day for the instrumented spinal fusion cohort. Subjects for the nonspinal fusion cohort were also selected from the same period and database. These subjects who did not undergo spinal fusion surgery were defined as the control. The control subjects were individually matched at a ratio of 10:1 to those who had undergone instrumented spinal fusion for age, sex, and the index day. Those who had a previous closed fracture of the thoracic vertebra or closed fracture of the lumbar vertebra since the initiation of the NHI program were excluded. Comorbidities were classified as diseases such as diabetes mellitus, hypertension, osteoporosis, and cardiovascular disease that existed before the index day. The end of the follow-up period for the analyses was marked on the day that new VCFs developed, enrolment in the NHI was terminated, death, or until the end of 2012 (Figure 1).

### Statistical Analysis and Comorbidity Risk Analysis

Differences among the groups were evaluated using Student *t* test for continuous variables and the *χ*^2^ test for categorical variables. The Cox proportional hazard model was used to evaluate the risk of developing new VCFs between the instrumented spinal fusion cohort and nonspinal fusion cohort. The hazard ratio (HR) showed that the confidence interval (CI) was 95%, and the *P* value was 2-sided. All *P* values <0.05 were considered significant. A Cox proportional hazards regression model (stratified by age, sex, and comorbidities) was also used to estimate the risk of new VCFs. Sensitivity analyses were performed to examine whether the main findings met the various assumptions. These analyses were also performed using the Cox model on subgroups classified by age, sex, and comorbidities. The forest plot was used to show all sensitivity analyses. All data management and HR calculations were performed using the Statistical Analysis System software for Windows (version 9.3; SAS Institute, Cary, NC).

## RESULTS

**FIGURE 2:**
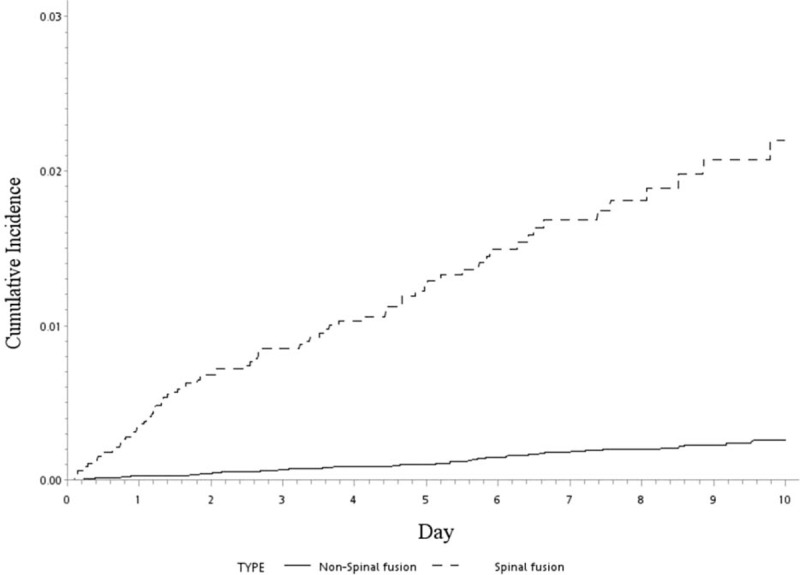
The cumulative incidence of closed fracture of thoracic vertebra or closed fracture of lumbar vertebra in Spinal fusion and non-spinal fusion cohorts.

**FIGURE 3:**
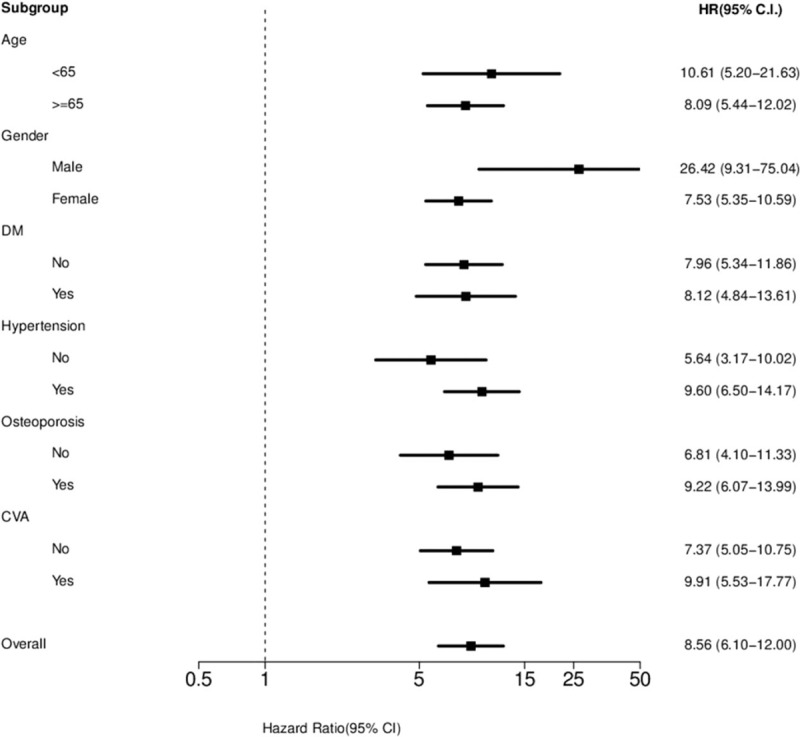
Forest plot of hazard ratios.

Overall, 76,439 patients were selected from the LHID 2000 between 1997 and 2012, divided into the spinal fusion and nonspinal cohorts, and reviewed. All patients were followed for 15 years in the NHRI database. The distributions of age and sex were not different between the 2 groups. The incidence of comorbidities was much higher in patients who had undergone spinal fusion than in those who had not undergone spinal fusion (*P* < 0.0001). Patients who had undergone spinal fusion surgery had a high incidence of developing new VCFs (1.87% vs 0.25%, *P* < 0.0001) (Table 1). The HR for these patients was 8.56 (95% CI, 6.10–12.00), which was adjusted for age, sex, and the index day. The cumulative risk of subsequent VCFs in the spinal fusion cohort increased over time (Figure 2). The age at each incremental year did not significantly increase the risk of new VCFs in the adjusted multivariate analyses. The presence of diabetes mellitus, hypertension, and cardiovascular disease also did not help predict new VCFs, but osteoporosis may have increased the risk of subsequent spinal fracture (HR: 1.56, *P* = 0.0324) (Table 2). The forest plot of HRs showed that young patients, male sex, and the presence of comorbidities were associated with high HRs (Figure 3).

**TABLE 1:**
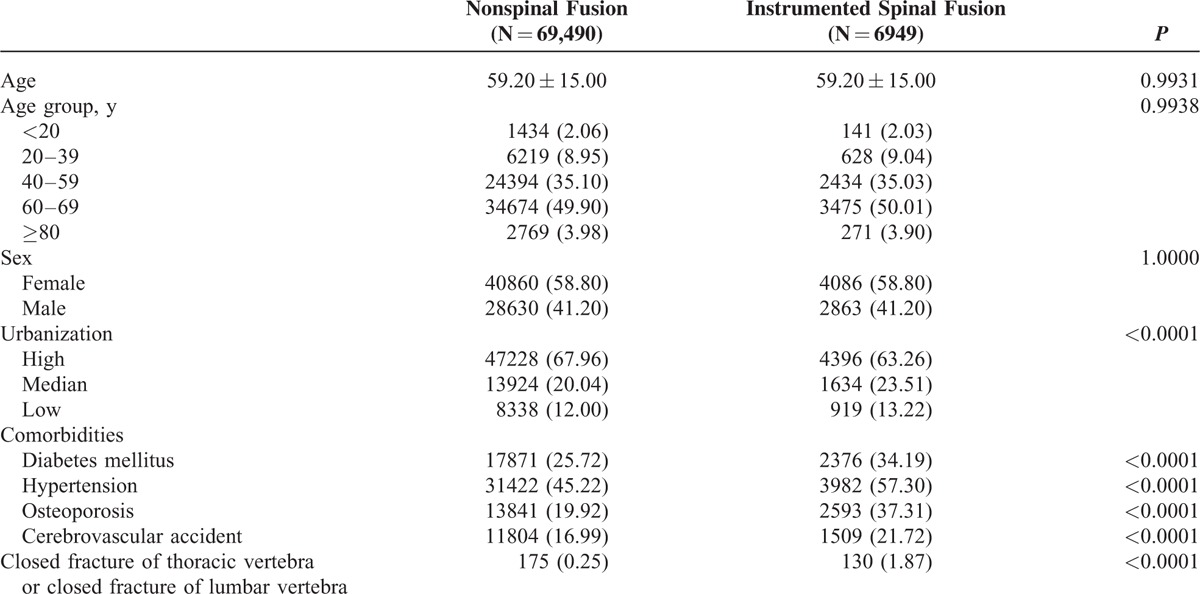
Characteristics of the Study Subjects

**TABLE 2:**
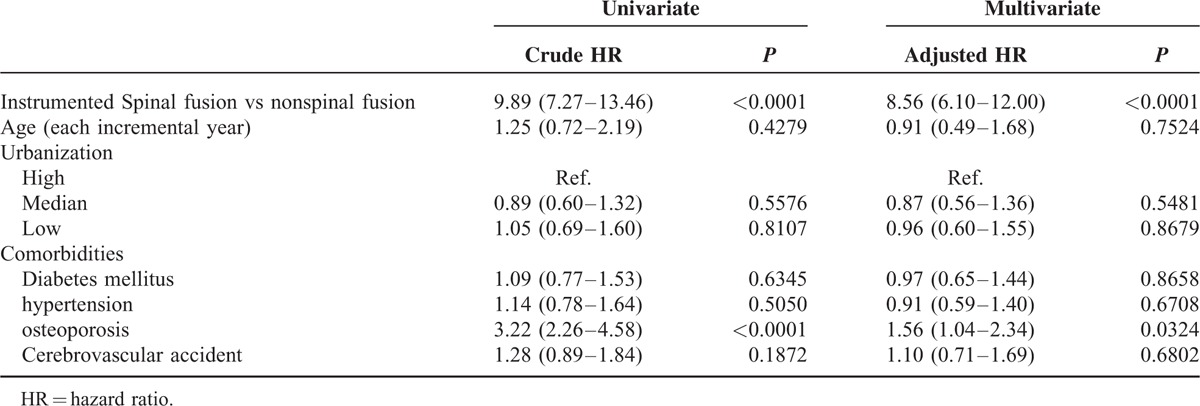
Univariate and Multivariate Analyses for Predicting Closed Fracture of Thoracic Vertebra or Closed Fracture of Lumbar Vertebra

### Differences Between the Young And Elderly Patients

Table 3 shows the HR of developing new VCFs for both cohorts stratified by age. Regarding the age-specific risk of closed spinal fracture between the spinal fusion and nonspinal fusion cohorts, the HR was higher in young patients than in elderly patients (10.61 vs 8.09). Comorbidities did not significantly increase the risk of new VCFs in both the young and elderly patients.

**TABLE 3:**
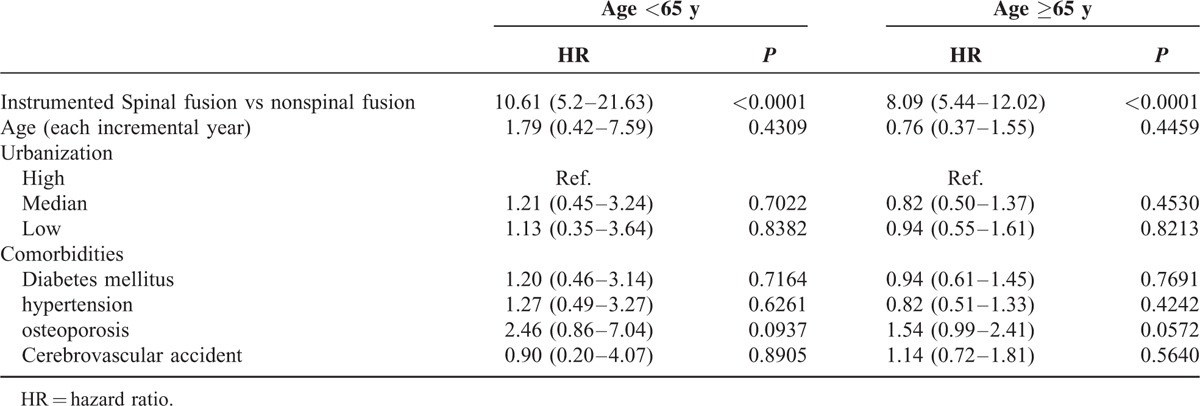
Univariate and Multivariate Analyses of Age for Predicting Closed Fracture of Thoracic Vertebra or Closed Fracture of Lumbar Vertebra

### Differences Between Men and Women

Table 4 shows the HR of developing new VCFs for both cohorts stratified by sex. Regarding the sex-specific risk of closed spinal fracture between the spinal fusion and nonspinal fusion cohorts, the HR was higher in men than in women (26.42 vs 7.53). In addition, the proportion of subsequent VCFs was much higher in men who had undergone spinal fusion than in men who had not undergone spinal fusion (1.01% vs 0.05%). However, women overall exhibited a more prevalent percentage of new VCFs than men (Table 5). The presence of osteoporosis significantly increased the risk of developing new VCFs in both sexes.

**TABLE 4:**
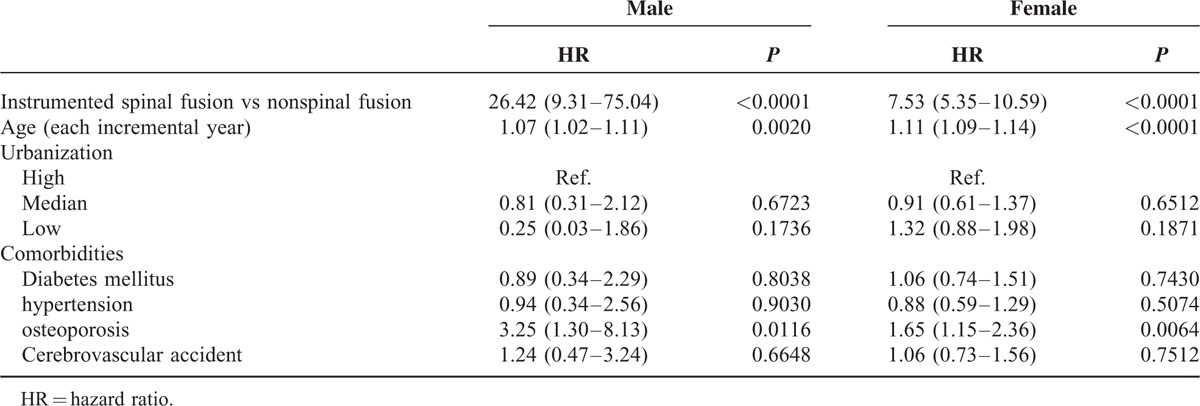
Univariate and Multivariate Analyses of Sex for Predicting Closed Fracture of Thoracic Vertebra or Closed Fracture

**TABLE 5:**
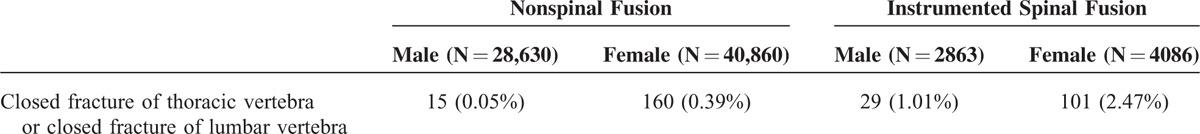
Cross Table by Gender and Event (Closed Fracture of Thoracic Vertebra or Closed Fracture of Lumbar Vertebra)

### Differences Between Thoracic and Lumbar Fractures

In this study, the locations of the fractures in the 2 groups were also identified by closed fractures of the thoracic vertebra (*ICD-9-CM* code 805.2) and lumbar vertebra (*ICD-9-CM* 805.4) in the LHID 2000 from 1997 to 2012. A summary of the locations of the fractures is as follows: thoracic vertebra fracture in 63 subjects, lumbar vertebra fracture in 156, and a nondifferential fracture location (including both the thoracic vertebra and lumbar vertebra) in 86 (Table 6). Table 7 shows the multivariate analyses of the risks for closed fractures stratified by location. The incidence of developing new VCFs was significantly increased in patients who had undergone spinal fusion surgery regardless of the location (adjusted hazard ratio [aHR] = 4.89 for thoracic VCFs, aHR = 3.76 for lumbar VCFs, aHR = 79.87 for thoracic and lumbar VCFs).

**TABLE 6:**
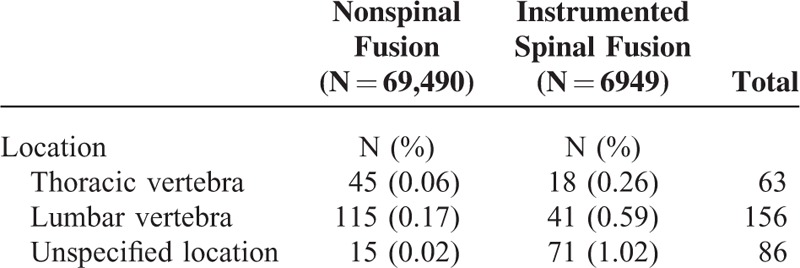
The Distribution of Subjects with New Vertebral Compression Fractures

**TABLE 7:**
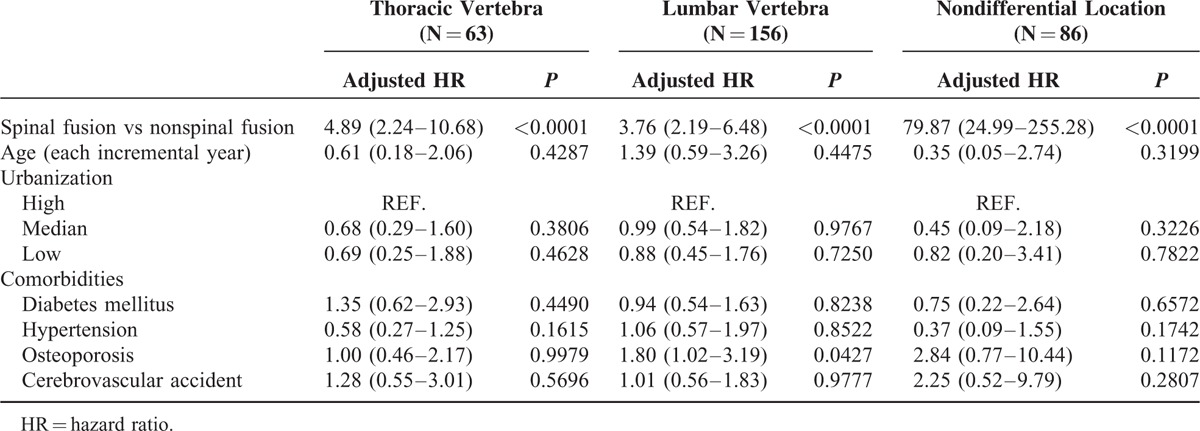
Multivariate Analyses of Risks for Closed Fractures Stratified by Locations

## DISCUSSION

VCFs occur when a block-like part of an individual vertebra becomes compressed because of trauma. The development of VCFs is usually related to osteoporosis, and they can cause severe back pain. The increasing incidence of subsequent VCFs after vertebroplasty or kyphoplasty has been reported in the literature with a subsequent fracture rate of 12% to 52%.^16,17^ Fribourg et al conducted a retrospective review of 38 patients who underwent kyphoplasty. Ten patients sustained 17 subsequent vertebral fractures over the follow-up period (average, 8 months). Of the 17 subsequent fractures, 9 occurred at the adjacent-above levels, 4 at the adjacent-below levels, and 4 at remote levels.^18^ Grados et al^16^ retrospectively reviewed 25 patients with 34 vertebras treated by vertebroplasty, and they showed a slight but significantly increased risk of vertebral fracture in the vicinity of cemented vertebras (odds ratio, 2.27; 95% CI, 1.1–4.56). Chen et al^19^ retrospectively reviewed 106 consecutive patients who underwent percutaneous vertebroplasty for osteoporotic VCFs, and they found that cement leakage into the disk can significantly increase the risk of adjacent vertebral fracture. However, some authors have mentioned that the subsequent VCF risk was not increased after vertebroplasty.^20,21^ Hence, the real relationship between vertebroplasty and subsequent VCFs is still unclear. Although it is still debatable, there is the possibility that spinal rigidity may increase after vertebroplasty, which can increase the risk of subsequent VCFs. In instrumented spinal fusion, at least 2 vertebras are fixed with instrumentation, which can create a new longer and rigid segment. Theoretically, the risk of subsequent VCFs in the vicinity of the vertebras may be increased. However, the relationship between developing subsequent VCFs and instrumented spinal fusion has not been well defined. To the best of our knowledge, our study is the first to address the long-term risk of subsequent VCFs after instrumented spinal fusion surgery using a retrospective cohort.

In our previous study, we retrospectively reviewed 1936 patients who underwent instrumented spinal fusion surgery. New subsequent VCFs occurred in 224 patients (11.6%). Of those, 150 patients were in the circumferential fusion group and the other 74 were in the PLF group. The overall new VCF rates were nearly equal between these 2 groups (11.1% and 12.5%, respectively). PLF alone with instrumentation was sufficient to cause a high risk of subsequent VCFs. The add-on use of posterolateral interbody fusion did not further increase the incidence of VCFs.^22^ Toyone et al retrospectively analyzed 100 consecutive patients 55 years or older who underwent spinal fusion for degenerative diseases. Acute VCFs were found in 15 patients (15%) during the mean follow-up period of 10.2 years (range, 7–14 years).^23^ In our study, the incidence of new VCFs after instrumented spinal fusion surgery was 1.87%. The incidence was significantly higher than that in the nonfusion group (0.25%, *P* < 0.001), but it was much lower than that found in previous studies.^22,23^ According to a literature review, only one-third of patients with VCFs will present with intractable back pain.^24^ In addition, low back stiffness with a foreign body sensation during outpatient follow-up is a common symptom and sign of patients who have undergone instrumented spinal fusion surgery. Therefore, the diagnosis of new VCFs after spinal fusion surgery may be underestimated because it is considered a normal occurrence.

A decrease in vertebral bone mineral density is usually noted after spinal fusion surgery. Bogdanffy et al reported that the bone mineral density of vertebra at 1 and 2 levels above the fusion segment significantly decreased at 3 months postoperatively. The bone density remained decreased until 6 months postoperatively. The authors attributed this phenomenon to postoperative immobilization, biomechanical alteration after fusion, and bone remodeling.^15^ Mcaffee et al conducted an animal model to observe bone remodeling after spinal instrumentation. They found that the rigidity of spinal instrumentation led to device-related osteoporosis of the vertebra.^26^ Once osteoporosis develops, compensated hyperactivity of an unfused segment in activities of daily living may increase the risk of proximal VCFs. Axelsson et al studied 6 patients who underwent radiographic analysis of segment motion before and after fusion surgery. They found that fusion of the lumbosacral segment can alter the kinematics of the adjacent segment, redistributing mobility toward relative hypermobility in the juxtafused segment.^25^ The change in biomechanics may result in a higher incidence of proximal VCFs. Hence, it is not surprising that the incidence of subsequent VCFs increases after instrumented fusion surgery.

The risk factors for proximal compression fracture after spinal fusion have been widely discussed. Female sex, old age, osteoporosis, treatment with interbody fusion or long segment fusion, and global sagittal imbalance have been considered risk factors.^27–30^ In a retrospective study of 125 cases in 1999, the risk of subsequent VCFs was clearly high in those who underwent lumbar fusion with rigid fixation. The risk appeared to be extremely high in postmenopausal women.^31^ In our study, the incidence of new and subsequent VCFs was high in elderly and female patients, which is comparable with previous studies’ findings. However, in particular, male and younger (<65 years) patients had higher HRs of new VCFs than female and elderly patients. A possible explanation for this was that the decrease of bone mineral density after spinal fusion surgery was more aggravated in patients with good bone quality preoperatively. Although a decrease in bone mineral density was also noted in osteoporotic patients, the effect was not as obvious as that in nonosteoporotic patients. This means that patients without osteoporosis may have a more increased risk of subsequent VCFs after spinal fusion surgery than those who have pre-existing osteoporosis. In our study, comorbidities were classified as those existing before the index day, and these included diabetes mellitus, hypertension, osteoporosis, and cardiovascular disease. There were no significant differences in the risks related to these confounding factors between the 2 study groups, except for osteoporosis. Although osteoporosis itself may increase the risk of subsequent VCFs, its effect on the increased risk of VCFs was not as high as in patients who underwent spinal fusion (HR: 1.56 vs 8.56). The development of new VCFs after instrumented spinal fusion should be monitored closely. Furthermore, the cumulative risk of new closed spinal fracture in patients who have undergone spinal fusion also increased over time.

The thoracic and lumbar vertebrae are different. First, the lumbar vertebrae are much larger than the thoracic vertebrae. Second, the vertebral body of the lumbar vertebrae is cylindrical or kidney-shaped, whereas that of the thoracic vertebrae is heart-shaped. Third, thoracic vertebrae articulate with the ribs and via the ribs to the sternum. Therefore, they are much less mobile and less likely to develop degenerative osteoarthritis. Fourth, zygapophyseal joints between the articular facets of the thoracic vertebra are directly vertical, so they limit flexion and extension, but allow rotation. Fifth, the thoracic vertebrae give the thoracic spine a concave curvature anteriorly, whereas the lumbar vertebrae produce lordosis. All the aforementioned reasons may interfere with the incidence of developing new VCFs; thus, further evaluation is crucial. In our study, we also analyzed the locations of newly developing VCFs. The locations of VCFs were divided into thoracic, lumbar, and nondifferential. Regardless of the location, the incidence of developing new VCFs was significantly higher in the spinal fusion group than in the control group. There were no significant differences in the risks related to these confounding factors. Thus, the fracture risk increased simultaneously in both the thoracic and lumbar vertebrae after spinal fusion surgery regardless of the location.

The strength of this study was that uniform data collection was performed in a well-defined population. However, there are several limitations worth highlighting. First, the insurance dataset does not provide detailed information on physical activity, economic status, daily exercise, bone mineral density, habits, the long-term use of steroid therapy, hormone therapy, and patient compliance, which are all potentially confounding factors relevant to the development of subsequent VCFs. Although each group of patients should be equally affected by these comorbidities because of the large number of cases, selection bias may still exist in this kind of study. Second, the levels of fusion and VCFs could not be determined in the dataset. The long fusion level can result in higher stress over an adjacent segment, which may further increase the rate of adjacent VCFs. Third, a retrospective cohort study design is subject to biases associated with confounding adjustments. Despite using a carefully designed study with adequate controls, bias may have remained because of unmeasured or unknown confounders. Fourth, the standard diagnosis of a new VCF is difficult to make. In general, developing VCFs should be diagnosed by clinical symptoms and an imaging study (eg, radiography or magnetic resonance imaging). However, some VCFs may not be represented in the database because of missed coding by doctors or patients who were asymptomatic, which may further increase the bias in this study. Additional longitudinal studies are necessary to validate the relationship between spinal fusion surgery and subsequent VCFs. Despite the inherent limitation of large population-based studies, we think that the findings of this large-scale research study can represent the impact of instrumental spinal fusion surgery on developing new VCFs.

## CONCLUSIONS

The results of this retrospective cohort study indicated that the risk of new VCFs increased significantly after spinal fusion surgery. In particular, the HR was high in male patients and those younger than 65 years. Although instrumented spinal fusion surgery is widely used and the clinical outcomes are usually satisfactory, surgeons should remember that it is not a complication-free procedure. Better patient selection, adequate protection, and aggressive treatment of osteoporosis are key factors for reducing the risk of subsequent VCFs after spinal fusion surgery.

## References

[1] Knoeller SM Seifried C Historical perspective: history of spinal surgery. *Spine* 2000; 25:2838–2843. 11064533 10.1097/00007632-200011010-00020

[2] Christopher RG Evolution in the treatment of spinal deformity and spinal instrumentation. *J Spinal Res Found* 2010; 5 1:19–25.

[3] Harrington PR The history and development of Harrington instrumentation. *Clin Orthop Relat Res* 1973; 93:110–112. 4579094 10.1097/00003086-197306000-00013

[4] Luque ER The anatomic basis and development of segmental spinal instrumentation. *Spine* 1982; 7 3:256–259. 7112238 10.1097/00007632-198205000-00010

[5] Hu SS Pashman RS Spinal instrumentation evolution and state of the art. *Invest Radiol* 1992; 27 8:632–647. 1428742 10.1097/00004424-199208000-00015

[6] Cotrel Y Dubousset J Guillaumat M New and universal instrumentation in spinal surgery. *Clin Orthop Relat Res* 1988; 227:10–23. 3338200

[7] Wang JC Mummaneni PV Haid RW Current treatment strategies for the painful lumbar motion segment: posterolateralfusion versus interbody fusion. *Spine* 2005; 30 16:33–43. 16103832 10.1097/01.brs.0000174559.13749.83

[8] Wu CH Wong CB Chen LH Instrumented posterior lumbar interbody fusion for patients with degenerative lumbar scoliosis. *J Spinal Disord Tech* 2008; 21 5:310–315. 18600138 10.1097/BSD.0b013e318148b256

[9] Gertzbein SD Betz R Clements D Semirigid instrumentation in the management of lumbar spinal conditions combined with circumferential fusion. A multicenter study. *Spine* 1996; 21 16:1918–1926. 8875726 10.1097/00007632-199608150-00018

[10] Fourney DR Abi-Said D Lang FF Use of pedicle screw fixation in the management of malignant spinal disease: experience in 100 consecutive procedures. *J Neurosurg* 2001; 94 1:25–37. 11147865 10.3171/spi.2001.94.1.0025

[11] Fisher CG Sahajpal V Keynan O Accuracy and safety of pedicle screw fixation in thoracic spine trauma. *J Neurosurg Spine* 2006; 5 6:520–526. 17176016 10.3171/spi.2006.5.6.520

[12] Park P Garton HJ Gala VC Adjacent segment disease after lumbar or lumbosacral fusion: review of the literature. *Spine* 2004; 29 17:1938–1944. 15534420 10.1097/01.brs.0000137069.88904.03

[13] Chen WJ Lai PL Niu CC Surgical treatment of adjacent instability after lumbar spine fusion. *Spine* 2001; 26 22:519–524. 10.1097/00007632-200111150-00024 11707723

[14] Schlegel JD Smith JA Schleusener RL Lumbar motion segment pathology adjacent to thoracolumbar, lumbar,;1; and lumbosacral fusions. *Spine* 1996; 21 8:970–981. 8726202 10.1097/00007632-199604150-00013

[15] Bogdanffy GM Ohnmeiss DD Guyer RD Early changes in bone mineral density above a combined anteroposterior L4-S1 lumbar spinal fusion. A clinical investigation. *Spine* 1995; 20 15:1674–1678. 7482016 10.1097/00007632-199508000-00005

[16] Grados F Depriester C Cayroile G Long-term observations of vertebral osteoporotic fractures treated by percutaneous vertebroplasty. *Rheumatology(Oxford)* 2000; 39:1410–1414. 11136886 10.1093/rheumatology/39.12.1410

[17] Uppin AA Hirsch JA Centenera LV Occurrence of new vertebral body fracture after percutaneous vertebroplasty in patients with osteoporosis. *Radiology* 2003; 226:119–124. 12511679 10.1148/radiol.2261011911

[18] Fribourg D Tang C Sra P Incidence of subsequent vertebral fracture after kyphoplasty. *Spine* 2004; 29 20:2270–2276. 15480139 10.1097/01.brs.0000142469.41565.2a

[19] Chen WJ Kao YH Yang SC Impact of cement leakage into disks on the development of adjacent vertebral compression fractures. *J Spinal Disord Tech* 2010; 23 1:35–39. 20065868 10.1097/BSD.0b013e3181981843

[20] Klazen CA Lohle PN de Vries J Vertebroplasty versus conservative treatment in acute osteoporotic vertebral compression fractures (Vertos II): an open-label randomised trial. *Lancet* 2010; 376 9746:1085–1092. 20701962 10.1016/S0140-6736(10)60954-3

[21] Chen D An ZQ Song S Percutaneous vertebroplasty compared with conservative treatment in patients with chronic painful osteoporotic spinal fractures. *J Clin Neurosci* 2014; 21 3:473–477. 24315046 10.1016/j.jocn.2013.05.017

[22] Li YC Yang SC Chen HS Impact of lumbar instrumented circumferential fusion on the development of adjacent vertebral compression fracture. *Bone Joint J* 2015; 97 10:1411–1416. 26430018 10.1302/0301-620X.97B10.34927

[23] Toyone T Ozawa T Kamikawa K Subsequent vertebral fractures following spinal fusion surgery for degenerative lumbar disease: a mean ten-year follow-up. *Spine* 2010; 35 21:1915–1918. 20838274 10.1097/BRS.0b013e3181dc846c

[24] Riggs BL Melton LJ 3rd The worldwide problem of osteoporosis: insights afforded by epidemiology. *Bone* 1995; 17 5:505–511. 10.1016/8756-3282(95)00258-4 8573428

[25] Axelsson P Johnsson R Strömqvist B The spondylolytic vertebra and its adjacent segment: mobility measured before and after posterolateral fusion. *Spine* 1997; 22 4:414–417. 9055370 10.1097/00007632-199702150-00012

[26] McAfee PC Farey ID Sutterlin CE 1989 Volvo Award in basic science. Device-related osteoporosis with spinal instrumentation. *Spine* 1989; 14 9:919–926. 2781409 10.1097/00007632-198909000-00003

[27] Ahn Y Lee SH Vertebroplasty for adjacent vertebral fracture following lumbar interbody fusion. *Br J Neurosurg* 2011; 25 1:104–108. 20825286 10.3109/02688697.2010.508848

[28] Cheh G Bridwell KH Lenke LG Adjacent segment disease following lumbar/thoracolumbar fusion with pedicle screw instrumentation: a minimum 5-year follow-up. *Spine* 2007; 32 20:2253–2257. 17873819 10.1097/BRS.0b013e31814b2d8e

[29] Watanabe K Lenke LG Bridwell KH Proximal junctional vertebral fracture in adults after spinal deformity surgery using pedicle screw constructs: analysis of morphological features. *Spine* 2010; 35 2:138–145. 20081508 10.1097/BRS.0b013e3181c8f35d

[30] Cho KJ Suk SI Park SR Short fusion versus long fusion for degenerative lumbar scoliosis. *Eur Spine J* 2008; 17 5:650–656. 18270753 10.1007/s00586-008-0615-z PMC2367413

[31] Etebar S Cahill DW Risk factors for adjacent-segment failure following lumbar fixation with rigid instrumentation for degenerative instability. *J Neurosurg* 1999; 90 2:163–169. 10199244 10.3171/spi.1999.90.2.0163

